# Boron as a Molecular Architect of Host–Microbiome Symbiosis: Implications for Dysbiosis and Aging-Related Pathologies

**DOI:** 10.3390/life16050750

**Published:** 2026-05-01

**Authors:** George Dan Mogoşanu, Andrei Biţă, Ion Romulus Scorei, Mihai Ioan Pop, Ilie Robert Dinu, Dan Ionuţ Gheonea

**Affiliations:** 1 Drug Research Center, Faculty of Pharmacy, University of Medicine and Pharmacy of Craiova, 2 Petru Rareş Street, 200349 Craiova, Romania; george.mogosanu@umfcv.ro (G.D.M.); andrei.bita@umfcv.ro (A.B.); 2 Department of Pharmacognosy & Phytotherapy, Faculty of Pharmacy, University of Medicine and Pharmacy of Craiova, 2 Petru Rareş Street, 200349 Craiova, Romania; 3 Department of Biochemistry, BioBoron/Natural Research Institute, 31B Dunării Street, 207465 Podari, Romania; 4 Department of Biotechnologies in Pharmaceutical Industry, Faculty of Biotechnologies, University of Agronomic Sciences and Veterinary Medicine of Bucharest, 59 Mărăşti Avenue, Sector 1, 011464 Bucharest, Romania; mihai.pop24@bth.usamv.ro; 5 Department of Nephrology, Faculty of Medicine, University of Medicine and Pharmacy of Craiova, 2 Petru Rareş Street, 200349 Craiova, Romania; robert.dinu@umfcv.ro; 6 Department of Gastroenterology, Research Center of Gastroenterology and Hepatology, University of Medicine and Pharmacy of Craiova, 2 Petru Rareş Street, 200349 Craiova, Romania; dan.gheonea@umfcv.ro

**Keywords:** boron, microbiota-accessible boron, dysbiosis, host–microbiome interface, mucosal barrier, quorum sensing, autoinducer-2, inflammaging, aging-related pathologies, lipidomics

## Abstract

Boron (B) is increasingly recognized as more than a trace dietary element, emerging as a context-dependent organizer of molecular interactions at the host–microbiome interface. B exhibits reversible covalent chemistry driven by Lewis’ acidity and selective affinity for *cis*-diol-rich biomolecules, enabling dynamic complexation with polyols, glycans, and phenolic ligands that dominate the intestinal mucus environment and shape microbial ecology. We synthesize evidence supporting an architecture-based framework in which B modulates biological function by conditioning the physicochemical context of microbial communication rather than acting as a single-pathway effector. Central to this model is spatial bioavailability, distinguishing plasma-accessible boron from microbiota-accessible boron (MAB), species that persist in the lumen and mucus layer long enough to influence interface-level processes. We propose that insufficient or altered MAB availability may contribute to dysbiosis (DYS) by destabilizing quorum-associated coordination, signal persistence, and mucosal microstructure, thereby promoting barrier dysfunction and inflammaging. Particular attention is given to B-mediated symbiotaxis, a hypothesis-driven concept describing how B-containing molecular assemblies may bias microbial communities toward cooperative, barrier-supportive configurations and reduce ecological volatility. We identify key knowledge gaps and experimental priorities (speciation-aware measurements, signal-centric readouts) necessary to determine when, where, and how B-mediated molecular architecture may counteract DYS and support healthspan.

## 1. Introduction

### 1.1. Aging and Dysbiosis as Loss of Multiscale Biological Coherence

Aging is commonly framed as a gradual accumulation of molecular lesions (deoxyribonucleic acid (DNA) damage, proteostatic stress, mitochondrial dysfunction, and chronic inflammation) that collectively erode physiological function [1,2]. Alongside these damage-centric models, systems-level perspectives increasingly interpret aging as a progressive decline in biological coherence: a loss of coordination and communication fidelity across interacting networks spanning molecules, cells, tissues, and microbial communities. In this view, age-associated dysbiosis (DYS) and mucosal barrier dysfunction are not merely correlates but potential drivers of coherence decay, amplifying inflammatory noise and destabilizing metabolic control.

Recent syntheses converge on aging as a system-level phenomenon emerging from the progressive decoupling of biological subsystems rather than isolated molecular damage alone [3,4]. Central to this decoupling is the host–microbiome (HM)–mucosal barrier axis, which integrates metabolic, inflammatory, and signaling coherence across spatial scales and may function as a control hub for resilience during aging and in DYS-linked pathologies [5]. Conceptually, coherence-based framings cast senescence as an entropic drift toward reduced coupling among regulatory loops (metabolic, mitochondrial, neurocognitive, and chronobiological), thereby lowering energetic efficiency and functional integration [6].

The gut microbiome (GM) provides a particularly salient context for this coherence framework. In early life and adulthood, host physiology and microbial ecology behave as a coupled system supported by reciprocal signaling through metabolites, immune calibration, and mucosal barrier dynamics [7]. With advancing age, many cohorts show reproducible shifts in microbiome structure and function, including reduced diversity, altered short-chain fatty acid (SCFA) production, enrichment of pathobionts in susceptible individuals, and increased inter-individual variability, changes that associate with frailty, metabolic dysregulation, and low-grade inflammation (“inflammaging”) [8,9,10,11]. These shifts frequently map onto clinically relevant phenotypes (metabolic syndrome, insulin resistance, metabolic dysfunction-associated fatty liver disease (MAFLD), cardiometabolic disease, and neuroinflammatory trajectories), supporting the view that DYS operates as a pathophysiological amplifier rather than a passive marker.

Importantly, these features are not merely compositional; they often reflect altered network behaviors, including perturbed trophic interactions, weakened community stability, and degraded signal integration. This motivates a mechanistic question central to healthspan biology: *which molecular factors help preserve or destabilize HM coherence as aging progresses?*

Interventional studies, including dietary modulation and fecal microbiota transplantation, support the view that age-associated microbial states can be causal contributors to systemic aging phenotypes rather than simple correlates. Yet many approaches remain largely outcome-driven, describing shifts in taxa or biomarkers without resolving the architectural mechanisms that stabilize or destabilize microbial coordination and signal integration at the mucosal interface.

### 1.2. Why Boron Is Relevant Beyond “Trace Element” Thinking

Boron (B) is traditionally discussed in nutrition as a trace element with heterogeneous evidence regarding essentiality in humans [12]. Yet B has an unusual chemical profile that makes it mechanistically relevant to biological organization. As a trivalent Lewis acid, B forms reversible covalent interactions with *cis*-diols and related nucleophiles, enabling transient complexation with polyols, glycans, and phenolic compounds—molecular classes that dominate the HM interface (diet-derived polyphenols, mucin (MUC) oxygen (O)-glycans, microbial and host glycoconjugates). This chemistry suggests that B can influence biological function through structure-mediated mechanisms: stabilizing, reorganizing, or buffering reactive molecular ensembles rather than operating through a single receptor–ligand pathway [12]. Accordingly, B chemistry is positioned to influence microbial stability vs. DYS through interface-level effects on mucus-associated molecular organization and communication.

Several lines of evidence support the plausibility of B as a modulator of inflammatory tone, redox balance, and metabolic signaling, although causal mechanisms remain incompletely defined. Experimental and clinical observations have associated B exposure or B-containing complexes with changes in inflammatory markers, oxidative stress parameters, and selected metabolic endpoints [13]. Mechanistically, B’s reversible interactions with hydroxyl-rich metabolites raise the possibility that B can influence redox-sensitive networks and methylation-linked physiology indirectly, through modulation of metabolite availability, compartmentalization, and reactivity [14]. These considerations align with the broader thesis of this review: *B may contribute to resilience by participating in the molecular architecture that supports HM coupling*.

### 1.3. Boron at the Host–Microbiome Interface: From Bioavailability to Accessibility

A key limitation in conventional discussions of B physiology is the implicit assumption that biological relevance scales primarily with systemic absorption (“bioavailability”) [13]. While circulating B species are undoubtedly important, HM interactions are also shaped by the fraction of B that reaches, persists, or is generated within the intestinal lumen and mucus layer, where microbial metabolism, quorum sensing (QS), and barrier interactions occur. This motivates a DYS-relevant functional distinction between plasma-accessible boron (PAB; systemically absorbed species) and microbiota-accessible boron (MAB; luminal or mucosa-associated B species capable of participating in microbial or interface-level chemistry) [12]. The latter may include B transiently complexed with dietary polyols and phenolics or borate species associated with MUC glycans and microbial exopolysaccharides. Importantly, “accessibility” here is not a synonym for poor absorption; it is a mechanistic category describing where B chemistry is positioned to influence communication and ecological dynamics.

Within this framework, diet becomes a critical determinant. Foods rich in polyols and polyphenols can expand the repertoire of B-complexed molecular forms, potentially altering their persistence through digestion and their availability to the colon [15]. Conversely, low-B intake and diets that reduce complex-forming substrates may constrain B-dependent chemistry at the HM interface. These considerations are particularly relevant in aging, where dietary patterns, mucosal glycosylation, and microbial enzymatic capacity can shift in ways that reshape the chemical landscape of the gut [16].

### 1.4. A Mechanistic Anchor: Boron-Dependent Quorum Sensing Chemistry

One of the most compelling mechanistic intersections between B and microbial ecology involves the QS autoinducer-2 (AI-2) molecule, a widely conserved signal implicated in interspecies communication. In certain bacterial systems, the biologically active form of AI-2 is a borate diester, linking B availability to the structural identity of a QS signal [17]. This provides a concrete example of how B can act as a structural determinant of microbial communication. Because AI-2 networks are implicated in community stability, biofilm ecology, and inflammation-associated shifts, AI-2–borate (AI-2B) chemistry offers a plausible bridge between B speciation and DYS dynamics. While the extent to which AI-2B chemistry is limiting in vivo across human gut environments remains an open question, AI-2B provides a valuable model: B can influence ecological coordination by controlling the stability and functionality of specific signaling architectures. This mechanistic anchor motivates a broader, hypothesis-driven concept: B may support the orientation of microbial communities toward cooperative equilibria by stabilizing chemical forms that enable coordinated behavior. We refer to this organizing idea as B-mediated symbiotaxis, a proposed role for B-containing molecular assemblies in shaping the spatial and functional alignment of microbial and host processes within the gut ecosystem [18]. Here, “symbiotaxis” is used as an integrative descriptor rather than a claim of established causality; it frames testable predictions about how MAB species could influence community stability, mucosal resilience, and inflammatory tone.

A particularly well-characterized example of B involvement in microbial communication is AI-2B, identified in specific bacterial systems such as *Vibrio* sp. This molecule illustrates that B can directly participate in molecular recognition processes relevant to QS [19].

However, the quantitative relevance and distribution of AI-2B in human GM remain incompletely defined. In this context, AI-2B should be considered a proof-of-principle for B-mediated signaling rather than a universal mechanism operating across all microbial communities.

More broadly, B may influence quorum-associated processes not only through specific ligands such as AI-2B but also by modulating the stability, availability, and spatial distribution of signaling molecules within chemically heterogeneous microenvironments.

### 1.5. Scope and Aims of This Review

In this review, we synthesize evidence from chemical biology, microbiome ecology, and aging research to evaluate B as a candidate molecular architect of HM symbiosis. Specifically, we (*i*) summarize aspects of B chemistry most relevant to biological interfaces; (*ii*) introduce an accessibility-based framework for B bioavailability that distinguishes systemic and microbiota-facing pathways; (*iii*) examine B-linked mechanisms of microbial communication, with emphasis on quorum-associated chemistry; and (*iv*) discuss how interface-level effects could translate into systemic outcomes relevant to metabolism, immune regulation, and resilience to aging. We conclude by outlining key knowledge gaps and experimental directions needed to determine when, where, and how B-mediated molecular architecture can be leveraged to support healthspan.

A central integrative goal of this review is to propose that biological relevance in B physiology depends strongly on spatial localization and chemical form. By distinguishing PAB from MAB, we argue that B biology cannot be reduced to systemic absorption or circulating levels alone. Instead, the HM–mucosal interface is positioned as an architectural hub where reversible B–*cis*-diol chemistry may shape molecular organization, signal persistence, and microdomain structure in ways that influence microbial coordination and barrier-associated resilience. In this view, B is framed not as a classical micronutrient acting primarily through discrete systemic targets, but as a context-sensitive modulator whose effects may emerge most strongly at critical interfaces where localization, ligand chemistry, and interaction density converge.

Within this interface-centered framework, we further aim to contextualize emerging lipidomic and metabolic aging signatures as systemic readouts of HM organization rather than isolated downstream biomarkers. Accordingly, we reframe aging not primarily as a consequence of cumulative molecular damage, but as a progressive loss of communicative coherence across interacting biological systems, including metabolic, immune, microbial, and barrier-associated networks. This perspective provides a mechanistic bridge linking microbiome dynamics, mucosal integrity, metabolite signaling environments, and organism-level aging trajectories, and it motivates speciation-aware and pathway-aware experimental approaches to determine when B-mediated molecular architecture can meaningfully support healthspan.

Despite growing interest in B biology, most discussions still treat B primarily as a systemic micronutrient whose relevance scales with absorption and circulating concentration. Here, we address this conceptual gap by distinguishing PAB from MAB and proposing B-mediated symbiotaxis as a mechanistic framework linking B chemistry to HM coherence, DYS susceptibility, and aging-related resilience. In this review, B-mediated symbiotaxis is defined as the tendency of B-containing molecular assemblies to bias HM systems toward cooperative and barrier-supportive configurations by conditioning communication chemistry and interface architecture. This framing is directly relevant to DYS-linked pathologies, where altered communication, barrier fragility, and ecological volatility can act as upstream drivers of systemic inflammatory and metabolic drift.

### 1.6. Current Evidence, Hypothesis Boundaries, and Testable Predictions

The framework proposed in this review integrates chemical, microbiological, and systems-level perspectives to explore B as a context-dependent modulator of HM organization. Given its interdisciplinary scope, it is important to explicitly distinguish between established evidence, biologically plausible mechanisms, and hypothesis-driven extrapolations.

At the level of established chemistry, B’s affinity for *cis*-diol-containing ligands and its ability to form reversible borate esters are well characterized. Recent work on microbial siderophores further supports the chemical plausibility of such interactions, showing that O-donor ligand systems, including catecholates and α-hydroxycarboxylates, can conditionally engage in reversible B coordination under specific environmental conditions, particularly in iron- and B-rich niches [20]. These interactions are known to occur with sugars, polyols, glycan structures, and phenolic compounds under physiologically relevant conditions [21]. In addition, the existence of B-containing signaling molecules, such as AI-2B identified in specific bacterial systems, demonstrates that B can participate directly in biologically meaningful molecular recognition processes [18,22].

However, the extension of these principles to the human gut ecosystem remains, in large part, a biologically plausible but not yet fully validated domain. In particular, the functional relevance of MAB as a distinct operational category [12,23], the in vivo significance of B–MUC interactions, and the extent to which B-dependent modulation of quorum-associated signaling contributes to microbial coordination in the human intestine all require direct experimental validation.

Accordingly, the concept of B-mediated symbiotaxis is presented here as a hypothesis-generating framework rather than a demonstrated physiological mechanism [18]. It provides a chemically grounded interpretation of how reversible B–*cis*-diol interactions could influence spatial organization, signal persistence, and ecological stability at the HM interface but does not presuppose causal confirmation in human systems.

This framework yields several experimentally testable predictions. These include: (*i*) the existence of distinct B speciation profiles within luminal and mucus-associated compartments compared to plasma [22]; (*ii*) measurable effects of B availability and ligand context on QS signal stability, including AI-2-related dynamics [24]; (*iii*) modulation of mucus microstructure, including viscosity, permeability, and microbial spatial distribution, under controlled B and ligand conditions [25]; and (*iv*) associations between altered MAB availability and markers of DYS, barrier dysfunction, and inflammaging [12,26].

Importantly, these relationships are expected to be bidirectional. Age-associated changes in mucus composition, microbial enzymatic activity, and dietary patterns may themselves influence B speciation and accessibility, thereby reshaping the chemical landscape of the HM interface.

By explicitly separating established evidence from hypothesis-driven interpretation, this section aims to provide a transparent conceptual boundary while preserving the integrative value of the proposed framework. In this context, B-mediated symbiotaxis should be understood as a testable organizing principle that links chemical form, spatial localization, and biological function across scales.

To further operationalize the conceptual framework, key terms used throughout this review can be linked to experimentally accessible readouts. In this context, microbiome coherence refers to the stability and reproducibility of community-level functions, including metabolite output consistency, reduced ecological volatility, and sustained coordination of microbial behaviors. Symbiotaxis refers to the tendency of HM systems to organize toward cooperative, barrier-supportive configurations, which may be reflected in spatial organization, communication fidelity, and resilience after perturbation. Information catalysis describes the stabilization and propagation of chemically labile, communication-relevant molecular states, such as quorum-associated signals or transient ligand assemblies, leading to improved signal persistence and interpretability within structured microbial communities.

These associations do not imply strict one-to-one mappings but rather provide an operational guide for translating conceptual constructs into experimentally testable variables.

## 2. Search Strategy and Selection Criteria for a Structured Narrative Review

This review was conducted as a structured narrative review designed to integrate representative evidence across B chemistry, HM interactions, and biological aging. Because the topic is interdisciplinary and hypothesis-generating, our aim was not to perform an exhaustive systematic review or quantitative meta-analysis but rather to identify and synthesize literature with strong mechanistic, conceptual, and translational relevance to the proposed framework.

### 2.1. Literature Search Strategy

A structured literature search was performed across PubMed/MEDLINE, Web of Science, and Scopus, complemented by targeted searches in Google Scholar to capture interdisciplinary and emerging publications that may not yet be fully represented in conventional indexing databases. Searches covered the period from January 2000 to January 2026, with particular emphasis on the last 10–12 years, reflecting the rapid expansion of microbiome, mucosal biology, and aging research.

Search terms were used in various combinations and included, but were not limited to, “boron chemistry”, “borate complexes”, “*cis*-diol interactions”, “microbiota-accessible boron”, “host–microbiome interface”, “mucus layer”, “quorum sensing”, “autoinducer-2”, “AI-2–borate”, “microbiome aging”, “dysbiosis”, “inflammaging”, “epigenetic drift”, “lipidomic aging signatures”, and “biological coherence”. Reference lists of key primary studies and authoritative reviews were also manually screened to identify additional relevant literature.

### 2.2. Selection Criteria

Studies were selected if they met one or more of the following criteria: (*i*) they provided mechanistic insight into B chemistry relevant to biological systems, including reversible B–*cis*-diol interactions and borate complexation; (*ii*) they addressed microbial communication, coordination, or ecological organization, particularly in relation to quorum-associated signaling or mucus-associated niches; (*iii*) they examined HM interactions related to barrier integrity, metabolic coupling, or immune modulation; or (*iv*) they contributed to systems-level or coherence-based conceptualizations of aging.

Both experimental and theoretical studies were considered, including in vitro, in vivo, ex vivo, and in silico investigations, as well as high-quality narrative reviews when these provided authoritative synthesis or conceptual advances. Studies focused exclusively on B toxicity, agricultural applications lacking biological interface relevance, and descriptive taxonomic surveys without functional or mechanistic interpretation were not prioritized for inclusion.

### 2.3. Data Integration and Interpretive Scope

Rather than quantitative aggregation, the selected literature was integrated through conceptual and mechanistic synthesis. Particular attention was given to chemical form, spatial localization, and interaction context, with the aim of linking molecular interactions to ecological organization and systemic aging phenotypes. This review therefore adopts an explicitly interpretive scope: it evaluates existing evidence, identifies hypothesis boundaries, and develops testable predictions regarding B-mediated organization at the HM interface.

Accordingly, the present work should be understood as a transparent, hypothesis-oriented narrative synthesis rather than a formal systematic review. Its purpose is to organize existing evidence across disciplinary boundaries, clarify where direct support currently exists, and identify experimentally tractable directions for future research.

## 3. Unique Chemical Properties of Boron Relevant to Biological Systems

### 3.1. Lewis Acidity and Reversible Covalent Chemistry

B occupies a singular position in biological chemistry due to its electron-deficient valence configuration. As a trivalent element with an incomplete octet, B functions as a classical Lewis acid, readily accepting electron pairs from nucleophilic donors, most prominently O atoms arranged in *cis*-diol geometries. This property sharply distinguishes B from transition metals and redox-active micronutrients that dominate conventional nutritional paradigms [27].

Unlike metal ions that typically engage in strong coordination complexes or irreversible catalytic centers, B–O interactions are intrinsically dynamic. Borate ester bonds form and dissociate reversibly in response to local pH, hydration state, dielectric constant, and ligand competition. This conditional chemistry allows B to participate in transient molecular architecture rather than fixed enzymatic or structural roles, positioning it as a context-dependent chemical modulator rather than a classical cofactor [28].

In aqueous biological systems, B exists primarily as boric acid (BA; B(OH)_3_), in equilibrium with tetrahedral borate species (B(OH)_4_^–^). This equilibrium is exquisitely sensitive to local chemical environments, enabling B’s biological relevance to emerge from spatial and contextual constraints rather than from concentration alone. As emphasized by Hunt (2012), such behavior places B outside classical micronutrient frameworks: its actions do not rely on enzymatic incorporation or redox cycling but on structural modulation of hydroxyl-rich molecular environments [29].

### 3.2. Boron as an Informational Catalyst

Within the broader logic of life’s chemistry, B can be viewed as an informational catalyst: a chemical agent that stabilizes fragile molecular states without irreversibly fixing them. During prebiotic evolution, B played a critical role in stabilizing ribose, the sugar backbone of ribonucleic acid (RNA), by forming borate esters that prevented rapid decomposition in aqueous environments. This selective stabilization enabled the emergence of RNA-based informational polymers and positioned B as an early arbiter of chemical order [30,31,32,33].

This catalytic logic persists in modern biological systems. By forming transient complexes with biological polyols and phenolic compounds, B generates localized chemical architectures that support higher-order organization without rigidification. In the gut environment, B can interact with MUC glycans, dietary polysaccharides, and microbial metabolites, creating a dynamic network of exchangeable complexes that stabilize the mucosal interface and modulate microbial adhesion and signaling [22,26,34]. In this sense, B functions less as a signaling molecule and more as a guardian of chemical coherence, preserving the fidelity of molecular communication in highly dynamic biological milieus.

*Cis*-diol motifs are among the most abundant functional groups in biological systems, occurring ubiquitously in mono- and oligosaccharides, glycoproteins, glycolipids, ribose-containing nucleotides, and a wide spectrum of dietary polyphenols. B displays pronounced affinity for these motifs, forming five- or six-membered cyclic borate esters under physiologically relevant conditions [35].

Crucially, B–*cis*-diol interactions do not merely result in passive sequestration. Borate ester formation can alter ligand solubility, conformational flexibility, redox potential, and enzymatic accessibility—effects that are subtle yet systemically relevant in heterogeneous biochemical environments such as the gastrointestinal (GI) tract. At glycan-dominated interfaces, particularly the intestinal mucus layer, this chemistry modifies the chemical landscape in which signaling and metabolism unfold, shaping emergent system-level behaviors.

The chemical features distinguishing B from classical micronutrients, together with their implications for mucosal integrity and inflammaging, are summarized in Table 1.

### 3.3. Boron–Polyol and Boron–Phenolic Complexes in Biological Contexts

Diet-derived polyols and polyphenols constitute a major reservoir of *cis*-diol-containing molecules encountered by the HM system. Numerous plant metabolites, including sugar alcohols, flavonoids, catechols, and chlorogenic acid derivatives, form thermodynamically stable B complexes under physiological conditions [23,28].

Such complexes are frequently favored over free BA and may persist across multiple compartments of the digestive tract, particularly when embedded within complex food matrices. These assemblies challenge the traditional view of B as a freely diffusible micronutrient, suggesting instead that a substantial fraction of dietary B functions as ligand-bound molecular entities [36].

Biologically, this has two major implications. First, complexation decouples systemic absorption from local chemical activity, allowing B to exert interface-specific effects independent of plasma levels. Second, B–polyphenol assemblies may exhibit emergent properties—altered redox behavior and modified interactions with microbial enzymes—that are not predictable from either component alone. Among these, diester chlorogenoborate (DCB) represents a particularly informative example. Resistant to gastric hydrolysis, DCB can reach the colon intact and function as an MAB structure. Experimental and conceptual work suggests that such complexes may modulate microbial metabolism and support AI-2B-dependent quorum signaling, positioning B as a mediator of microbial communication rather than a passive dietary element [23,26,34,37].

These complexes exemplify B’s ecological adaptability: through reversible covalent bonding, B links dietary polyphenols with microbial and host molecules, acting as a chemical currency of symbiosis.

### 3.4. Interaction with Glycans and Mucosal Architecture

The intestinal mucus layer is a glycan-dense hydrogel composed primarily of heavily O-glycosylated MUCs, functioning simultaneously as a physical barrier, biochemical filter, microbial habitat, and signaling interface [38]. *Cis*-diol motifs are abundant throughout MUC glycans, rendering the mucus matrix chemically compatible with reversible B interactions [39].

At present, however, direct in vivo evidence demonstrating stable or functionally significant B–MUC complexes in the human intestine remains lacking. Accordingly, the relevance of such interactions should currently be regarded as chemically plausible and biologically suggestive rather than directly established in physiological settings.

This plausibility arises from first principles of B coordination chemistry. The high density of *cis*-diol-containing glycans within MUCs, together with the hydrated and dynamically structured nature of the mucus gel, creates an environment in which transient borate-mediated interactions could occur. Even if such interactions are weak, reversible, and spatially heterogeneous, they may still influence mesoscale properties of the mucus layer, including local ordering, hydration, porosity, diffusion behavior, and microbial spatial organization.

Importantly, these proposed effects do not require the existence of long-lived stoichiometric B–MUC complexes. Rather, they may emerge from distributed, low-frequency coordination events operating within a crowded glycan-rich matrix. In this sense, B is hypothesized to act as a contextual modulator of mucus-associated chemical organization rather than as a conventional structural crosslinker [22].

This hypothesis remains experimentally tractable. Glycan-rich and polymer-based hydrogel systems are known to exhibit emergent structural and diffusional properties under reversible chemical interactions [40]. Future studies could test it using ex vivo mucus systems, purified MUC hydrogels, synthetic glycan-rich matrices, and spatially resolved B speciation analyses under gut-relevant physicochemical conditions. Such approaches would help determine whether B measurably alters mucus microstructure or HM spatial organization in biologically meaningful ways.

### 3.5. Structural Modulation vs. Receptor-Driven Signaling

A defining feature of B biology is the absence of a dominant receptor-mediated signaling paradigm. Unlike classical hormones, vitamins, or trace elements, B does not exert its influence primarily through high-affinity protein targets. Instead, its effects emerge through structural modulation of molecular ensembles [36].

This mode of action aligns with contemporary systems biology perspectives emphasizing weak multivalent interactions, contextual regulation, and mesoscale organization as determinants of biological function. By stabilizing glycan-rich matrices, modulating polyphenol chemistry, and influencing the persistence and spatial distribution of signaling molecules, B operates as a chemical architect rather than a molecular messenger, a role particularly relevant in the gut ecosystem, where collective behaviors arise from dense networks of low-affinity interactions.

### 3.6. Implications for Host–Microbiome Systems

Taken together, B’s Lewis acidity, selective *cis*-diol affinity, and reversible covalent chemistry provide a coherent mechanistic basis for its proposed role at the HM interface. These properties enable B to participate in transient structural networks that integrate dietary compounds, microbial metabolism, and host-derived glycans into a chemically coherent system.

Rather than acting as a discrete signal or nutrient in the classical sense, B contributes to the chemical scaffolding that supports biological resilience, adaptability, and coherence in the gut environment [34]. This perspective reframes B bioavailability in functional terms, emphasizing where and how B acts rather than how much is absorbed systemically.

**Figure 1:**
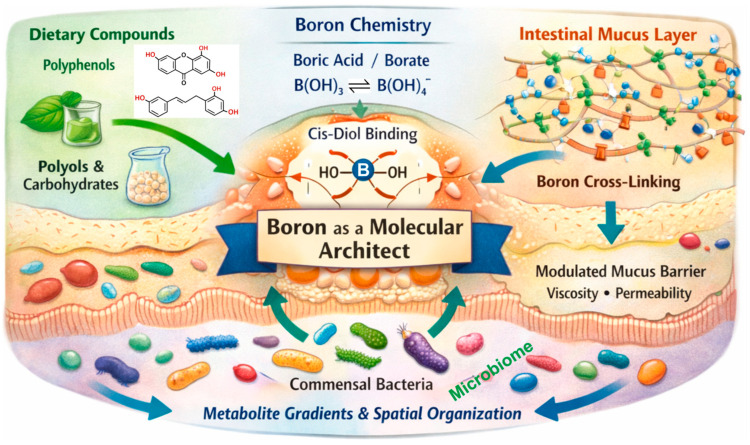
B as a molecular architect at the HM interface. B’s Lewis acidity and selective affinity for *cis*-diol-containing molecules enable reversible covalent interactions with dietary polyphenols, polyols/carbohydrates, and glycan-rich mucosal components. Rather than acting primarily through receptor-mediated signaling or enzymatic catalysis, B participates in transient molecular assemblies that condition the physicochemical organization of the intestinal mucus layer. Through these weak, context-dependent interactions, B can modulate mesoscale properties such as mucus viscosity, permeability, and spatial structuring, thereby shaping microbial localization and metabolite gradients at the mucus-adjacent interface. Collectively, these interface-level effects support HM coherence and functional resilience. The figure integrates the chemical principles summarized in Table 1 into a systems-level conceptual model of HM symbiosis. Conceptual illustration created by the authors. B: Boron; HM: Host–microbiome.

These principles are summarized in Figure 1, which frames B as a context-dependent molecular architect operating within the MUC–glycan matrix at the HM interface. This chemical perspective shifts attention away from B as a purely systemic micronutrient toward its localized, form-dependent functions at biological boundaries. It also sets the stage for the following section, where PAB is distinguished from MAB and where spatial accessibility, rather than systemic absorption alone, is argued to define functional relevance in B biology.

## 4. Boron Bioavailability Revisited: Plasma-Accessible vs. Microbiota-Accessible Boron

### 4.1. Why “Absorption” Is Not Synonymous with “Biological Relevance”

Conventional frameworks in B nutrition have largely equated biological relevance with systemic absorption and plasma concentration. This approach reflects classical micronutrient paradigms developed for elements whose primary actions involve enzymatic cofactors, redox cycling, structural incorporation, or endocrine regulation. Consequently, B has been evaluated predominantly through metrics of absorption efficiency, urinary excretion, and safety margins, yielding essential kinetic and toxicological insight [28,29].

However, a plasma-centric definition of bioavailability is incomplete for elements whose principal actions may occur at biological interfaces, where chemistry governs organization and communication rather than receptor-bound signaling. The GI tract is not a passive conduit for uptake but a chemically active ecosystem in which dietary ligands, microbial metabolism, and host secretions continuously reshape local molecular architectures. In such settings, the fraction of B that remains luminal or associates with the mucosal interface may exert effects that are independent of, or antecedent to, systemic distribution.

This logic parallels a key reframing in microbiome science: for microbiota-accessible carbohydrates, functional relevance is defined not by host absorption but by colonic accessibility and ecological consequence [41,42]. By analogy, B biology requires an explicit distinction between systemic exposure and interface-level chemical accessibility, particularly given B’s reversible interactions with polyols, glycans, and phenolic compounds. The implication is conceptual but testable: “bioavailability” is not a single scalar variable but a spatially structured phenomenon whose biological meaning depends on localization and chemical form.

This framework also helps explain why single-ingredient or static microbiome interventions often yield inconsistent outcomes, particularly in aging populations. Effective modulation may require ecosystem-level strategies that stabilize communication architectures and interface organization, rather than merely altering component abundance.

### 4.2. Plasma-Accessible Boron: Established Systemic Pathways

PAB denotes B species that are absorbed across the intestinal epithelium and distributed systemically [12]. Under typical dietary conditions, B is efficiently absorbed largely as BA and small borate species, readily detected in plasma and urine, forming the basis for most nutritional and toxicological assessments.

Systemic B exposure has been associated with effects across mineral metabolism, inflammatory markers, cognitive performance, and selected endocrine-related endpoints, although mechanistic specificity remains limited and often indirect. In contrast to canonical micronutrients, B does not appear to act via a dominant high-affinity receptor or an obligatory enzymatic cofactor role; rather, reported effects are commonly framed in terms of context-dependent modulation of inflammatory tone, redox balance, and related regulatory milieus [28].

A key limitation of this literature is an implicit assumption: because BA is rapidly absorbed, the unabsorbed fraction is treated as biologically negligible. This inference presupposes that biological relevance scales linearly with plasma levels, an assumption increasingly challenged in systems where interface chemistry and microbial ecology act as upstream determinants of inflammation and resilience.

### 4.3. Microbiota-Accessible Boron: A Functional Category

To address these conceptual limitations, we propose MAB as a functional category distinct from plasma-accessible forms. MAB refers to B species that persist within the intestinal lumen, mucus layer, or immediate HM interface long enough to participate in local chemical, structural, and ecological processes [23,26].

Crucially, MAB is defined by accessibility rather than absorption efficiency. It includes B transiently complexed with dietary polyols, phenolic compounds, and glycan-rich host secretions, as well as B embedded within food matrices that delay, redistribute, or spatially restrict absorption. This builds on earlier proposals that B can behave as a prebiotic-like chemical factor, influencing biological systems through reversible molecular interactions rather than direct nutrient signaling [36,43].

Importantly, MAB does not imply poor absorption or nutritional inadequacy. Instead, it reflects dynamic partitioning of B between systemic and interface-level compartments. Factors shaping this partitioning include dietary composition and food structure, GI pH gradients, mucosal glycosylation patterns, hydration and ionic context, and microbial enzymatic activity. In this sense, MAB may constitute a quantitatively smaller B pool, yet one that is plausibly positioned to influence HM coordination by conditioning signal persistence, spatial organization, and barrier-adjacent chemistry [12].

The operational distinctions between PAB and MAB [12], including localization, dominant chemical forms, measurement strategies, and functional implications, are summarized in Table 2. Together, these distinctions emphasize that “bioavailability” is not a single scalar variable but a spatially structured phenomenon with distinct readouts and mechanistic meaning.

Because MAB is defined by spatial accessibility and chemical persistence rather than by systemic absorption alone, its experimental study requires dedicated compartment-specific and speciation-aware strategies.

### 4.4. Experimental Considerations for MAB Detection and Quantification

A major limitation of the MAB framework is that it is currently easier to define conceptually than to measure directly. Unlike PAB, which can be approximated through total B measurements in plasma or urine, MAB requires compartment-specific and speciation-aware approaches capable of resolving B within the intestinal lumen, mucus layer, or immediate HM interface.

At the operational level, MAB should not be inferred solely from total B intake or systemic exposure. Rather, its measurement would ideally distinguish free BA/borate from ligand-bound, matrix-associated, or mucus-associated B species that persist locally long enough to influence interface-level chemistry. This creates an analytical challenge because B complexes are expected to be reversible, environmentally sensitive, and potentially altered during sample collection and processing [44].

Several experimental strategies may help address this problem. First, compartment-resolved sampling could compare B speciation across plasma, luminal contents, and mucus-associated fractions in gut-relevant model systems or animal studies. Second, speciation-aware analytical approaches, including ^11^B–nuclear magnetic resonance (NMR), B-sensitive liquid chromatography–mass spectrometry (LC–MS) workflows, and orthogonal chromatographic separation of B–ligand complexes, may help distinguish free from complexed forms under controlled conditions [45,46]. Third, ex vivo mucus systems, purified MUC hydrogels, and synthetic glycan-rich matrices could be used to test whether B partitions preferentially into mucus-like environments or alters their structural and diffusional properties [47].

These approaches also have important limitations. B complexes may undergo exchange during dilution, pH shifts, freezing, or extraction; mucus turnover and microbial metabolism can rapidly alter local chemistry; and direct access to human gut mucus microdomains is inherently difficult. For these reasons, the first goal of MAB-oriented experimentation may not be absolute quantification but rather the demonstration of differential localization, persistence, and ligand-dependent partitioning under biologically relevant conditions.

Accordingly, MAB should presently be viewed as an experimentally tractable but not yet standardized category. Its value lies in directing attention toward spatially resolved B chemistry at the HM interface and in motivating the development of analytical frameworks that go beyond total systemic B measurements.

### 4.5. Dietary Determinants of MAB and Distal-Gut Boron Chemistry

Diet is a primary determinant of B speciation and fate in the GI tract. Plant-derived foods rich in polyols and polyphenols provide abundant *cis*-diol ligands capable of forming borate esters under physiologically relevant conditions. Relative to free BA, ligand-bound forms may display altered solubility, hydrolytic stability, and compartmental persistence, increasing the likelihood that a fraction of ingested B remains available beyond the upper GI tract.

Food processing, matrix structure, and co-ingested nutrients further modulate these interactions. Fiber-rich matrices can physically retain B-containing assemblies and slow their dissolution and uptake, while microbial fermentation can release, transform, or redistribute ligand-bound species within the distal gut. In this sense, MAB is not merely a chemical entity but an emergent dietary outcome, shaped by the coupling of ligand chemistry (polyols/polyphenols), physical structure (plant matrices and fiber), and microbial metabolism.

Accordingly, dietary patterns emphasizing minimally processed plant foods may favor MAB generation and interface-level persistence, whereas refined diets low in complex carbohydrates and polyphenols may bias B kinetics toward rapid absorption and systemic clearance [12,23]. This aligns with a broader principle from microbiome science: chemical form and physical context, not total intake alone, determine microbial accessibility and ecological consequences.

### 4.6. Spatial Partitioning of Boron and Interface-Level Accessibility

The functional distinction between PAB and MAB can be understood primarily as a problem of spatial partitioning. Conventional nutritional assessments capture the systemic compartment reasonably well because rapidly absorbed B species are reflected in plasma concentrations and urinary excretion. However, this view underrepresents a second biologically relevant compartment in which B remains localized within the intestinal lumen, mucus layer, and immediate HM boundary.

Within this interface-facing compartment, B may persist through reversible complexation with dietary polyols, polyphenols, fiber-associated ligands, and glycan-rich mucosal components. This does not imply strong or permanent binding. Rather, it suggests a pool of chemically labile but spatially retained B species that may remain locally available long enough to influence microenvironmental conditions, ligand exchange, and interaction opportunities at the mucosal frontier.

This distinction matters because biological relevance may depend not only on the extent of systemic absorption but also on whether B remains chemically accessible within the distal gut long enough to participate in local chemical processes. In this sense, MAB should not be interpreted simply as “unabsorbed B” but as a functionally distinct pool defined by localization, chemical form, and persistence within the mucus–lumen continuum. Analogous behavior has been observed in environmental systems, where B coordination with organic ligands occurs as a reversible and context-sensitive process, with binding equilibria shifting dynamically in response to local physicochemical conditions [20].

**Figure 2:**
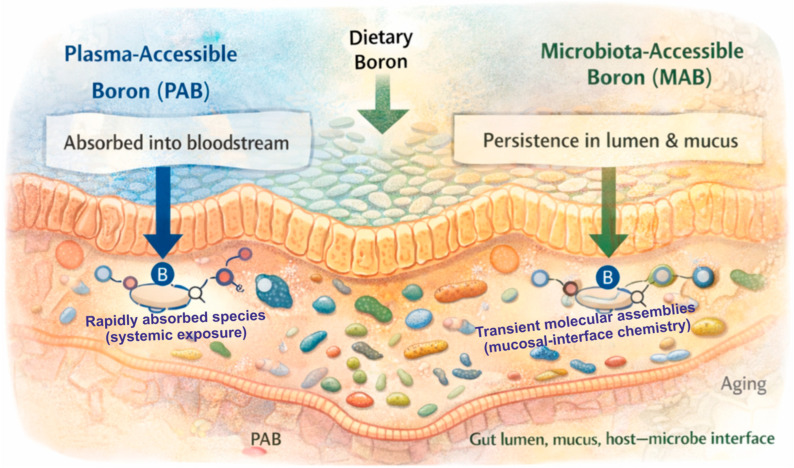
Spatial partitioning of B bioavailability in the gastrointestinal tract. Dietary B partitions into two biologically distinct pools defined by spatial accessibility and chemical form. PAB comprises B species, predominantly BA and small borates, that are rapidly absorbed and distributed systemically, supporting conventional systemic endpoints. In contrast, MAB refers to B species that persist within the intestinal lumen, mucus layer, and immediate HM boundary through reversible complexation with dietary polyols, polyphenols, fiber-associated ligands, and glycan-rich mucosal components. This distinction emphasizes that B “bioavailability” depends not only on absorption efficiency but also on local chemical accessibility within the mucus–lumen continuum. Aging-related changes in gastric acidity, mucosal structure, and microbiome composition may shift the balance between these compartments, with potential implications for barrier function and inflammaging. Conceptual illustration created by the authors. B: Boron; BA: Boric acid; HM: Host–microbiome; PAB: Plasma-accessible boron; MAB: Microbiota-accessible boron.

Aging is expected to influence this partitioning. Age-associated changes in gastric acidity, intestinal transit, mucus composition, hydration state, and microbial enzymatic activity may alter the balance between rapidly absorbed B species and those that remain available at the mucosal interface. This provides a rationale for considering B biology in explicitly spatial terms and supports the need for compartment-resolved, speciation-aware experimental approaches. Figure 2 summarizes the spatial partitioning of dietary B into plasma-accessible and microbiota-accessible compartments along the GI tract, providing a structural basis for distinguishing systemic exposure from lumen- and mucus-facing chemical interactions.

### 4.7. Aging, Microbiome Drift, and the Loss of Interface Coherence

Aging-associated gut DYS is increasingly understood not only as a taxonomic shift but as a progressive loss of ecological resilience, functional redundancy, and regulatory coordination within microbial communities [48,49]. In younger or more resilient ecosystems, cooperative guilds help maintain metabolic flexibility, support SCFA production, reinforce mucosal barrier function, and constrain inflammatory escalation [50,51]. With age, these community-level properties may erode, producing a more volatile relationship between microbial communities and host tissues [52,53].

In this review, we interpret this process as a form of microbiome drift, meaning a gradual decline in the fidelity and stability of community-level behaviors that normally sustain HM homeostasis. This drift may involve less reliable metabolite production, weaker cross-feeding structure, impaired stress–response coordination, reduced colonization resistance, and greater susceptibility to proinflammatory outputs [54,55,56]. Importantly, such deterioration may emerge before major taxonomic collapse becomes evident in conventional compositional datasets [57,58,59,60].

This perspective is relevant to the MAB framework because the mucosal boundary is not merely a passive surface but a chemically and spatially organized zone in which signal persistence, ligand exchange, diffusion constraints, and microbial positioning may shape ecological outcomes. If aging destabilizes this boundary, then local chemistries that help preserve organization at the HM frontier may become functionally important even in the absence of large systemic effects.

Within this context, MAB can be considered as a candidate contributor to local stability at the mucosal frontier. The possible mechanisms and experimental implications of this possibility are considered in the following section.

### 4.8. Conceptual Integration and Transition

Taken together, the distinction between PAB and MAB suggests that B bioavailability should not be reduced to systemic absorption alone [12]. A second dimension, local accessibility within the mucus–lumen continuum, may be equally important for understanding how B participates in HM organization.

Within this framework, aging-related microbiome instability can be interpreted not only as compositional change but also as a progressive loss of coordination at the mucosal frontier [60,61,62,63]. This perspective raises a mechanistic question: through which molecular routes could MAB influence microbial coordination, mucus-associated structure, and barrier-relevant ecological behavior?

The following section addresses this question by focusing on specific mechanistic classes and experimentally testable predictions rather than on broader conceptual re-statement.

## 5. Boron as a Molecular Architect of Host–Microbiome Symbiosis

### 5.1. Mechanistic Routes by Which Microbiota-Accessible Boron Could Stabilize Host–Microbiome Organization

The biological effects proposed here are unlikely to arise from a single receptor-mediated pathway. Rather, MAB is hypothesized to act through several overlapping physicochemical routes that influence how information, metabolites, and microbial behaviors are organized at the mucosal frontier.

One plausible route involves glycan- and mucus-associated interactions. Intestinal MUCs are densely decorated with *cis*-diol-containing glycans, creating a chemically compatible environment for reversible B coordination. Even weak and distributed B–diol interactions could alter local hydrogel properties such as hydration, porosity, diffusional constraints, and microdomain organization. A comparable form of context-dependent coordination has been described in microbial systems, where siderophore ligands with appropriate O-donor architectures can transiently bind B in a reversible manner, depending on metal availability, pH, and ligand environment, illustrating how such interactions may emerge as conditional physicochemical properties rather than dedicated biological functions [20]. Such effects would not require permanent bonding or stoichiometric crosslinking; relatively small changes in mesoscale organization may be sufficient to influence how microbes localize, compete, and interact at the mucus boundary.

A second route involves quorum-related communication chemistry, particularly within the LuxS/AI-2 signaling family. AI-2 is not a single static molecule but an ensemble of interconverting structures derived from 4,5-dihydroxypentane-2,3-dione (DPD), whose relative abundances are sensitive to local physicochemical conditions. In some systems, the biologically recognized ligand is a borated species, classically the LuxP-recognized furanosyl borate diester (AI-2B) [64,65,66]. This provides an important proof-of-principle: B can be embedded directly within a microbial communication ligand and can influence signal identity at the level of molecular geometry rather than functioning merely as a passive environmental factor.

At the same time, AI-2 perception is receptor-diverse. LuxP is only one sensing solution; many taxa rely on non-borated AI-2 forms or alternative sensing architectures, including LsrB-like systems and other receptor classes. AI-2B should therefore not be interpreted as a universal gut mechanism but as a stringent mechanistic precedent showing that B can participate directly in quorum-associated signaling where receptor context permits [67,68,69,70]. From this perspective, B may influence communication both directly, by stabilizing signal architectures that are biologically recognized, and indirectly, by shaping the local physicochemical milieu in which AI-2-related equilibria, transport, and reception occur [71,72,73].

These possibilities are especially relevant at mucosal surfaces, where signaling operates within spatially structured communities rather than in a homogeneous bulk phase. At this scale, modest changes in ligand stability, confinement, exchange, or diffusion may alter the lifetime and spatial footprint of communication cues. The gut can therefore be viewed as a mosaic of microdomains in which mucus association, dietary ligands, pH, ionic composition, and metabolite crowding may influence the balance between different AI-2-related forms and their ecological consequences.

A third route involves broader ecological biasing through changes in local signal persistence and microdomain chemistry. Because metabolites and signaling-relevant intermediates exist within structured gradients, modest effects on ligand stability or localization may scale into larger consequences for cooperation, cross-feeding, stress tolerance, and niche stabilization. In this sense, MAB is best viewed as a context-sensitive ecological modulator that may probabilistically favor cooperative, barrier-supportive configurations over unstable or inflammation-associated states [12].

These proposed mechanisms remain hypotheses, but they generate experimentally tractable predictions. If valid, MAB-enriched conditions should not necessarily produce dramatic taxonomic shifts. Instead, the strongest effects may appear in measures of community coordination, signal fidelity, barrier-adjacent organization, resilience after perturbation, and metabolite-network stability. This shifts the mechanistic emphasis from “which taxa increase” to “which local functions become more stable and coherent”.

### 5.2. Experimental Predictions and Validation Strategy

The symbiotaxis framework can be tested through progressively more tractable experimental layers. A first level would assess whether B differentially partitions into MUC-rich, glycan-rich, or fiber-rich environments relative to freely diffusible aqueous compartments. Ex vivo MUC hydrogels, synthetic glycan matrices, and controlled B–ligand systems could be used to quantify localization, persistence, and reversibility under gut-relevant pH and ionic conditions [74].

A second level would examine whether MAB alters mucus-associated structure or diffusion behavior. Relevant outputs include hydrogel permeability, retention of model metabolites or signaling ligands, and changes in the spatial organization of microbial consortia grown in mucus-like matrices or biofilm-compatible systems.

A third level would test quorum-related chemistry more directly. Gut-relevant microenvironmental conditions could be used to examine whether B availability shifts AI-2 speciation, signal persistence, or receptor-linked responses in taxa representing LuxP-, LsrB-, or other AI-2 sensing architectures [75,76]. Such studies would help distinguish direct signal-geometry effects from broader environmental modulation and clarify the degree to which B-dependent AI-2 architectures are relevant in mucus-associated communities.

A fourth level would map the ecological distribution of relevant sensing solutions across defined consortia or gnotobiotic systems. Particularly informative approaches would include identifying LuxP-, LsrB-, or double calcium channels and chemotaxis (dCACHE)-associated pathways in mucus-associated bacteria and testing whether MAB-enriched delivery shifts community behaviors consistent with improved coordination, barrier support, or recovery after perturbation.

Finally, animal or gnotobiotic systems could be used to determine whether MAB-enriched delivery alters functional readouts linked to HM stability, including SCFA consistency, barrier markers, inflammatory tone, ecological recovery after perturbation, and communication-linked community behavior. Importantly, the initial goal of validation is not to prove a universal B effect across all microbiomes but to determine whether B chemistry can measurably stabilize biologically relevant mucosal functions under defined conditions.

### 5.3. AI-2B as Proof-of-Principle for Boron-Dependent Microbial Communication

The LuxS-derived AI-2 system provides a rare, experimentally validated case in which B is not merely an environmental variable but an integral component of signal identity. Structural studies demonstrated that the LuxP receptor binds a furanosyl borate diester form of AI-2 (AI-2B), establishing that B can be embedded within a *bona fide* interspecies communication ligand [17]. In this context, borate complexation is not a minor chemical modification; it stabilizes a specific geometry required for receptor recognition and downstream signaling.

This precedent suggests two important implications for the symbiotaxis framework advanced in this review. First, it supports the possibility that B availability can, in principle, modulate microbial communication by biasing signal speciation, i.e., selecting which members of a dynamic equilibrium persist in a quorum-active conformation. Second, it motivates the broader hypothesis that reversible B chemistry can shape quorum-associated processes even when B is not obligatorily required for receptor binding by tuning signal lifetime, degradation kinetics, and spatial diffusion within structured microbial communities.

At the same time, an important boundary condition must be stated: the quantitative contribution of B-dependent AI-2 architectures in complex ecosystems such as the human gut remains incompletely defined [61]. Accordingly, AI-2B is treated here as a mechanistic anchor rather than a complete explanatory model [77]. Its primary value is conceptual and testable: it supports the possibility that B may increase communication fidelity by stabilizing signal geometry and persistence under microenvironmental constraints.

This proof-of-principle sets the stage for examining additional interface-level nodes, especially mucosal structuring and glycan-driven microdomain formation, through which B-containing assemblies may influence microbial ecology and host responses.

If AI-2B demonstrates that B can be “written into” a communication ligand, the next question is where such chemistry can plausibly operate at scale in vivo. We therefore turn to the mucus layer as an architectural substrate rich in *cis*-diol motifs, where B–*cis*-diol interactions could influence diffusion geometry, niche compartmentalization, and barrier-adjacent host signaling.

While AI-2B provides a chemically and structurally well-defined example of B participation in QS, its role in complex human-associated microbial communities remains to be established. Current evidence supports its function in specific bacterial taxa but does not yet demonstrate a dominant or universal role in the GM.

Accordingly, in the present framework, AI-2B is used as a mechanistic reference point illustrating how B can participate in intercellular signaling rather than as a generalizable model for all HM communication systems. Future studies employing speciation-resolved analytical approaches in gut-relevant environments will be required to determine whether AI-2–borate complexes contribute measurably to signaling dynamics in vivo.

### 5.4. Mucosal Interfaces as Sites of Boron-Mediated Organization

The intestinal mucus layer constitutes a primary arena for HM interaction, functioning simultaneously as a physical barrier, a selective ecological filter, and a nutrient/communication matrix. In the colon, secreted gel-forming MUCs, dominated by mucin 2 (MUC2), create a structured, hydrated network in which the inner mucus layer restricts bacterial access to the epithelium, while the outer layer supports a dense, spatially organized microbial habitat [78]. Extensive MUC-type O-glycosylation provides biochemical identity and functional diversity: MUC O-glycans act as adhesion substrates, ecological cues, and a conditional nutrient reservoir for mucus-associated consortia, particularly under fluctuating dietary input [79].

A critical point for the present framework is that mucus is not a uniform gel. Its viscoelastic properties, pore size, hydration state, and chemical composition vary across microdomains and along depth gradients, and these physical features shape bacterial localization, community structure, and barrier function [80]. Consistent with this, mucus rheology and viscosity gradients contribute to intestinal microbial biogeography, influencing proximity to the epithelium and exposure to host-derived antimicrobial factors [81]. This spatial structuring is biologically consequential because it governs the frequency of microbe–host contact, the effective diffusion of metabolites and signals, and the stability of cooperative niches at the mucosal surface [82].

Within this glycan-rich and spatially structured environment, reversible B chemistry becomes chemically plausible as an interface-level organizer [83]. MUC glycans present a dense landscape of polyhydroxylated motifs, and B (as BA/borate or within B-bearing complexes) can form reversible esters with *cis*-diols in a strongly context-dependent manner (local pH, ligand density, hydration state, and multivalency). While direct in vivo evidence for stable, long-lived B–MUC complexes remains limited, the mucus layer provides precisely the kind of crowded, multivalent glycan matrix in which transient borate-mediated associations could occur repeatedly and locally without requiring permanent crosslinking. In principle, such reversible interactions could modulate mucus microstructure by shifting local hydration, network cohesion, and microdomain organization, thereby altering the physicochemical constraints that govern microbial access, retention, and movement within the mucus gel [84].

From the symbiotaxis perspective, the central implication is not that B “locks” mucus into a rigid scaffold but that B may contribute to conditional stabilization: a tunable influence on the mucus-associated chemical milieu that shapes (*i*) the persistence and localization of microbial metabolites and communication-relevant ligands and (*ii*) the spatial distribution of taxa across mucosal niches. Because mucus-associated communities are often enriched in mutualistic, barrier-supportive guilds, and because MUC glycosylation patterns are tightly linked to host defense and tolerance strategies, even modest interface-level shifts in organization could plausibly translate into disproportionately large ecological effects [79].

In this context, the MAB framework yields a tractable and testable hypothesis: B delivered in forms that persist into the colon could bias interface conditions toward cooperative, mucosa-adapted community configurations while disfavoring taxa that benefit from barrier erosion or proximity-driven inflammation. This hypothesis predicts measurable outcomes under controlled B form and availability, including changes in mucus rheology and permeability, altered mucosal biogeography of key functional guilds, and shifts in the coupling between microbial signaling and epithelial responses.

Taken together, quorum-associated speciation and mucus microdomain organization motivate a unifying interpretation: B can influence HM systems by shaping the context in which coordination, niche structure, and barrier-adjacent mutualism emerge. We therefore integrate these threads into a hypothesis-driven framework (B-mediated symbiotaxis) and articulate testable predictions that distinguish architectural stabilization from composition-first or target-centric models.

### 5.5. Boron-Mediated Symbiotaxis: A Conceptual Framework

We propose B-mediated symbiotaxis as a conceptual framework describing how B-containing molecular assemblies may bias host and microbial systems toward cooperative, stable, and resilient configurations [18]. In this context, symbiotaxis does not denote a discrete molecular pathway or a single signaling axis. Rather, it refers to an emergent organizational tendency in which biological behaviors align through shared chemical and spatial constraints operating at the HM interface.

The core premise of this framework is that HM stability depends not only on community membership (“who is present”) but on the fidelity of interactions among community members and between microbes and host tissues (“how reliably they coordinate”). This interaction fidelity is shaped by microenvironmental features such as signal persistence, diffusion geometry, mucosal compartmentalization, and the physicochemical properties of glycan-rich matrices. Within this system-ecology view, B is positioned as a contextual modulator: through reversible interactions with *cis*-diol-containing substrates, B may tune the conditions under which communication, cooperation, and spatial niche structure emerge without acting as a classical receptor-specific effector.

Conceptually, B-mediated symbiotaxis can be understood through three interrelated dimensions. First, at the level of chemical stabilization, B participates in reversible complexation with glycans, polyols, polyphenols, and selected signaling intermediates. These interactions can alter local ligand stability, speciation, and residence time, thereby conditioning how chemical information persists and remains interpretable within crowded biological microdomains. In this sense, B contributes to signal and ligand conditioning rather than to direct signal generation.

Second, at the level of ecological orientation, modulation of signal persistence, diffusion constraints, and microdomain localization may bias coordination-dependent community behaviors, including quorum-associated transitions, cross-feeding, and mucus-adjacent niche occupation. The predicted outcome is not uniform across taxa but rather a probabilistic tendency to favor cooperative, mucosa-adapted guild configurations and to reduce ecological volatility that predisposes microbial ecosystems toward DYS.

Third, at the level of interface integration, the mucus layer and adjacent epithelium represent the principal substrate where host glycans, microbial metabolites, and dietary ligands converge. Through effects on glycan-rich matrices and the spatial organization of interface chemistry, B-mediated interactions may help maintain barrier-adjacent order, supporting separation between microbes and the epithelium while preserving the metabolic and signaling exchanges that underpin mutualistic HM relationships.

Importantly, this framework does not imply that B alone determines microbiome composition or host outcomes. Rather, symbiotaxis is presented as a hypothesis-driven organizing principle describing how a chemically subtle factor could exert system-level influence by shaping interaction context. Its value lies in generating falsifiable expectations: if B acts primarily as an architectural modulator, then biologically relevant effects should manifest as improvements in signal stability, spatial coherence, and recovery dynamics rather than solely as shifts in mean metabolite concentrations or taxonomic abundance.

**Figure 3:**
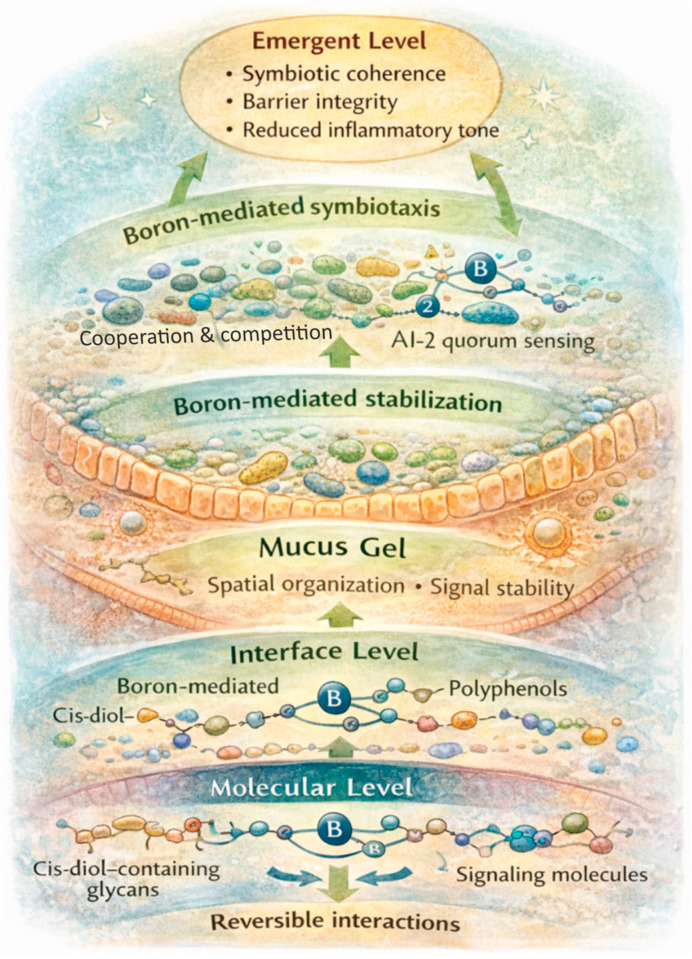
B-mediated symbiotaxis at the HM interface. This schematic depicts a multiscale model in which B participates in reversible interactions with *cis*-diol-containing glycans, dietary ligands (e.g., polyols and polyphenols), and selected signaling intermediates, contributing to the chemical architecture of the intestinal mucus layer. By conditioning molecular stability, spatial organization, and signal persistence within mucus-adjacent microdomains, B-containing assemblies can influence microbial coordination, niche occupation, and quorum-associated communication. These interface-level effects may propagate upward to emergent properties such as symbiotic coherence, barrier integrity, and reduced inflammatory tone, thereby providing an architecture-based mechanism for HM resilience. Conceptual illustration created by the authors. AI-2: Autoinducer-2; B: Boron; HM: Host–microbiome.

Accordingly, B form and accessibility are predicted to be critical determinants of outcome. MAB should exert effects distinct from PAB, even at equivalent total intake, by preferentially influencing mucus-associated microdomains, community coordination, and barrier-linked inflammatory variability. Furthermore, if aging reflects a progressive loss of communicative coherence, then B-mediated architectural stabilization would be expected to yield proportionally larger effects in aged or stressed systems, primarily by reducing biological noise, volatility, and fragility rather than simply lowering baseline biomarker values. Figure 3 schematically summarizes this multiscale framework, illustrating how reversible molecular interactions can scale upward through interface organization to produce emergent ecological and host-relevant outcomes. In this view, B-mediated symbiotaxis becomes an empirically testable, resilience-oriented model in which stability, variability, and spatial organization constitute primary outcome dimensions rather than magnitude-based biomarker shifts alone. This positions B symbiotaxis naturally within a geroscience framework, where aging is increasingly interpreted as system-level loss of resilience and coordination.

### 5.6. Boron Symbiotaxis in the Context of Geroscience: Aging as Loss of Symbiotic Coherence

The relevance of B-mediated molecular architecture to aging becomes most apparent when considered across levels of biological organization, from local chemical interactions to system-level regulation. Within the emerging framework of geroscience, aging is increasingly interpreted as a progressive loss of system-level resilience and coordination across interacting biological networks. As summarized in Table 3, the symbiotaxis framework predicts that B-sensitive effects may emerge first as changes in ligand conditioning and microdomain structure, then scale into community coordination and ecological resilience, and ultimately shape systemic inflammatory and metabolic trajectories.

Aging is increasingly associated with structural and functional alterations at the HM interface, including changes in MUC glycosylation, increased epithelial permeability in susceptible individuals, and shifts in microbial function and metabolic output [85]. Collectively, these features reflect a broader loss of symbiotic coherence in which communication and cooperation deteriorate across interconnected biological scales rather than failing at a single point.

Within this framing, reduced availability of microbiota-relevant B chemistry, whether due to low intake, altered chemical form, dietary contexts that limit colon delivery, or age-linked shifts in microbial metabolism, could plausibly bias ecosystems toward DYS-prone states and barrier fragility. Because many proposed B effects operate through signal conditioning and spatial organization, even modest perturbations could be amplified in aging systems where buffering capacity and adaptive flexibility are reduced.

Conversely, preservation of MAB chemistry may help sustain the structural conditions necessary for cooperative HM interactions. By supporting interface-level organization and reducing ecological volatility, B-mediated symbiotaxis could contribute to maintaining functional coherence under physiological stress and cumulative entropy. While direct causal evidence remains limited, this interpretation aligns B-mediated symbiotaxis with resilience-oriented definitions of aging biology, i.e., the capacity of biological systems to absorb perturbation while preserving integrated function, rather than with narrowly defined single-target anti-aging mechanisms.

Although these architectural roles operate primarily at interface and community levels, their consequences need not remain localized to the gut. By shaping microbial metabolism, barrier integrity, and immune signaling tone, interface stabilization can plausibly propagate into systemic metabolism and organism-level aging trajectories.

## 6. Systemic Implications: Metabolism, Immunoregulation, and Aging

### 6.1. From Interface-Level Architecture to Systemic Physiology

The architectural roles of B at the HM interface are not expected to remain confined to local ecological organization. Biological regulation is inherently hierarchical: coherence established at interface and community levels can propagate upward, shaping metabolic control, immune calibration, and inflammatory tone at the organismal scale. The gut therefore functions not only as a site of nutrient processing but as a regulatory hub whose outputs (microbial metabolites, barrier-derived signals, and immune cues) are continuously integrated by peripheral tissues.

Within this framework, B-mediated symbiotaxis can be interpreted as a systemic translation problem. When interface-level communication is stable and spatially organized, gut-derived signals are delivered in a relatively continuous, low-noise manner, supporting coherent host responses. Conversely, when mucosal architecture and microbial coordination deteriorate, stochastic fluctuations in metabolite flux, barrier permeability, and immune activation can be amplified and propagated systemically, contributing to chronic dysregulation over time.

Accordingly, B is not positioned here as a conventional pathway agonist acting through a single receptor-defined mechanism. Instead, it is framed as a contextual modulator whose physiological effects may emerge from changes in the quality, persistence, and integrability of gut-derived signals. This perspective predicts distributed and indirect influences across multiple domains rather than a linear, single-target causal chain.

### 6.2. Mechanistic Translation: From Interface Coherence to Host Resilience Pathways

Although B-mediated effects are proposed to operate primarily through interface-level architecture, their systemic relevance can be conceptualized through a translation mechanism: stable HM organization supports continuous, low-noise delivery of microbial metabolites and barrier-derived cues that converge on canonical resilience pathways. In this model, B is not treated as a direct agonist of adenosine monophosphate-activated protein kinase (AMPK), sirtuin 1 (SIRT1), or mechanistic target of rapamycin (mTOR). Rather, B is positioned as a contextual contributor to the reliability and interpretability of upstream inputs, particularly SCFA flux, bile acid signaling tone, redox-sensitive metabolites, and immune-calibration cues [86].

When interface coherence is preserved, these inputs are more likely to remain temporally stable and spatially structured, supporting robust host integration. When coherence is lost, fluctuating metabolite delivery, variable permeability, and unstable immune activation thresholds can amplify systemic variability and stress signaling, pushing homeostatic pathways toward compensatory dysregulation. Thus, the expected relationship between B chemistry and host resilience is distributed and contingent: B-accessible chemistry, especially in microbiota-accessible forms (MAFs), may improve the conditions under which canonical pathways maintain homeostatic regulation during aging.

This translation view also reframes experimental priorities. If the primary effect is stabilization of upstream signal fields then intervention studies should quantify signal stability and variability, not only mean shifts in biomarkers. In other words, the core prediction is improved coherence of gut-derived inputs and reduced biological noise at the systemic level (Table 4).

### 6.3. Immunoregulation and Inflammatory Tone

Immune homeostasis is tightly coupled to the structural and chemical integrity of the mucosal barrier, which controls microbial proximity, antigen exposure, and immune activation thresholds [87]. The mucus layer and epithelium are not passive boundaries; they function as an active regulatory interface that shapes tolerance, calibrates innate immune tone, and limits the probability that microbial-derived cues trigger disproportionate inflammatory responses. Disruption of these structures, through altered mucus organization, increased permeability, or shifts in mucus-associated community structure, can promote chronic low-grade inflammation and immune dysregulation, features frequently linked to metabolic impairment and aging.

From the symbiotaxis perspective, B-mediated molecular architecture may support immunoregulation by stabilizing interface organization and conditioning the persistence, diffusion geometry, and localization of microbial metabolites and signaling molecules within barrier-adjacent microdomains. By contributing to conditional stabilization in glycan-rich matrices, B-containing assemblies could bias the interface toward controlled immune calibration, lowering the likelihood that transient perturbations escalate into sustained inflammatory amplification. This does not require B to act as an immunosuppressive agent; rather, it implies a role in maintaining the physicochemical conditions under which immune tolerance and proportional responsiveness are more likely to be preserved.

Reports linking B status or supplementation to inflammatory biomarkers are compatible with this conceptual model, but key uncertainties remain. Directionality and dietary confounding must be addressed, and mechanistic work is needed to determine whether observed changes reflect barrier stabilization, altered microbiome functional outputs, or systemic effects independent of the gut. These limitations reinforce the need for integrative study designs that measure mucosal integrity, microbiome function, and inflammatory endpoints within the same experimental framework.

Given the tight coupling between barrier-linked inflammation and oxidative stress, the next mechanistic question is whether B-mediated interface stabilization can also condition redox-sensitive signaling networks, thereby modulating the amplification of inflammaging through nuclear factor erythroid 2-related factor 2 (Nrf2)-linked and related stress–response pathways.

### 6.4. Aging as Progressive Loss of Systemic Resilience

Aging is increasingly understood as a decline in the capacity of biological systems to maintain coherent function under stress [88]. This loss of resilience often manifests as increased variability in metabolic control, heightened inflammatory responsiveness, and reduced adaptability to environmental perturbations. Importantly, such features can emerge before overt disease, consistent with a view of aging as network instability and impaired signal integration rather than a sequence of single-point failures.

Within this resilience framing, B’s potential relevance lies not in acting as a classical anti-aging effector but in its proposed capacity to stabilize interface-level processes that buffer systemic physiology. If B-mediated symbiotaxis helps maintain organized microbial outputs, barrier integrity, and low-noise communication at the mucosal surface, while also supporting signal-centric rescue of community coordination (microbiome epigenetic rescue agents (MERA) logic), then it could plausibly attenuate the amplification of stochastic fluctuations that characterize aging systems. In this sense, B chemistry (particularly in MAFs) would be expected to influence not only mean biomarker levels but the volatility and coherence of age-related trajectories.

This framing avoids overstatement: it does not imply that B “prevents aging” but that B-accessible chemistry may modulate the conditions under which HM integration remains stable as resilience declines. Such an effect would be expressed most clearly in aged or stressed systems, where buffering capacity is reduced, through decreased inflammatory noise, improved barrier-linked tolerance, and more coherent metabolic signaling.

### 6.5. Context Dependence and Individual Variability

The systemic expression of B-mediated symbiotic organization is expected to be inherently context-dependent. Multiple layers of biological and environmental variation, including dietary matrix composition, microbiome functional capacity, baseline B status, age-associated changes in MUC glycosylation, and host genetic background, are likely to influence both the partitioning between PAB and MAB and the extent to which interface-level architectural effects translate into systemic outcomes.

Dietary context is particularly influential. Diets rich in polyphenols, polyols, and fermentable fibers increase the probability that ingested B enters microbiota-accessible chemical states capable of persisting within the mucus-adjacent environment [89]. In contrast, refined diets low in complex carbohydrates and phytochemicals may bias B kinetics toward rapid absorption and clearance, limiting interface-level engagement. These dietary effects are further modulated by microbiome functional traits, including MUC-degrading capacity, polyphenol metabolism, and QS competence, features that vary substantially between individuals and across life stages [90].

Age-related changes introduce additional layers of variability. Alterations in MUC glycosylation patterns, reduced mucus thickness in susceptible individuals [91], and shifts in microbial community structure can all reshape the chemical and spatial landscape in which B-mediated interactions occur. As a result, similar B intake levels may produce markedly different biological effects in younger vs. older hosts or in resilient vs. fragile physiological states.

This context dependence underscores the limitations of uniform intake metrics and static dose–response models. Instead, a resilience-oriented framework requires integration of chemical form, accessibility, localization, and interaction context. Operationally, this implies that translational and clinical efforts should prioritize measurable contextual features, such as dietary polyphenol and fiber patterns, microbiome functional readouts, mucosal barrier markers, and signal variability metrics, alongside total B exposure. Such designs are more likely to capture the conditions under which B-mediated symbiotaxis emerges as a biologically meaningful influence.

### 6.6. Summary: Resilience-Oriented Interpretation

Taken together, the systemic implications discussed in this section support a resilience-oriented interpretation of B biology. Rather than acting as a classical micronutrient defined primarily by systemic absorption and discrete metabolic targets, B is better conceptualized as a contextual modulator whose biological relevance emerges from reversible chemistry at critical biological interfaces, most notably the HM–mucosal axis.

Through this role, B-mediated symbiotaxis provides a coherent conceptual bridge linking chemical interactions and spatial organization in the gut to microbiome ecology and, ultimately, to organism-level regulation relevant to aging. The emphasis shifts from isolated pathways and single biomarkers toward the preservation of communication fidelity, spatial order, and ecological stability, features increasingly recognized as central to healthspan and aging resilience.

This interpretation also reframes how B-centered interventions should be evaluated. If B primarily influences resilience through interface-level architecture, then its most consistent effects are expected when (*i*) B chemical form is microbiota-accessible, (*ii*) mucus-associated organization and barrier integrity are measurable, and (*iii*) outcomes are assessed using integrated readouts that capture microbial function, barrier dynamics, and inflammatory–metabolic coherence within the same experimental framework. Variability, signal stability, and spatial organization thus become as informative as mean changes in classical biomarkers.

In this light, B-mediated symbiotaxis does not represent a narrowly defined anti-aging mechanism but a systems-level principle: subtle chemical stabilization at biological interfaces can reduce noise, preserve coordination, and slow the erosion of resilience that characterizes aging. This perspective positions B not as a universal solution but as a chemically grounded contributor to the maintenance of symbiotic coherence across the lifespan.

## 7. Knowledge Gaps and Future Directions

Interest in B biology has expanded rapidly, yet the field still faces a familiar problem: we often discuss B as if it were a single entity, while its biological effects likely depend on chemical form, localization, and context. This tension, between elegant hypotheses and limited in vivo resolution, defines the main knowledge gaps that must be addressed before concepts such as B-mediated symbiotaxis can be converted into quantifiable mechanisms, reproducible biomarkers, and rational intervention strategies. What follows is less a list of missing citations and more an agenda for how the field can become experimentally “honest” about where B is, what form it takes, and what it is realistically capable of doing at the HM interface.

### 7.1. In Vivo Chemical Speciation and Functional Boron Pools

The first and most fundamental gap is surprisingly basic: we still do not know what B “looks like” in vivo at the sites where it is hypothesized to matter most. Many studies report total intake, urinary excretion, or circulating concentrations, but these metrics do not reveal which molecular forms actually exist in the gut lumen, within mucus, or at the epithelial boundary. If B’s relevance is architecture-driven, through reversible complexation, then the key question becomes: *which B–polyol, B–glycan, or B–phenolic assemblies are present under physiological conditions, and which of these constitute a functionally relevant microbiota-accessible pool?* Progress here will require analytical strategies that can capture transient interactions without destroying them during sampling, coupled with metallomics and structural approaches that can distinguish free BA/borate from complexed species in biologically realistic matrices.

### 7.2. Spatial Localization, Microdomain Dynamics, and Temporal Resolution

Even if we identify B species, the next challenge is determine where and when these interactions occur. The chemistry central to this review is reversible and context-dependent, which means static measurements may miss the biologically meaningful “moments” of organization: microdomain formation, signal persistence, or local shifts in diffusion geometry. What is needed are time-resolved and compartment-specific approaches: mucus-focused sampling, chemical imaging, and integrated metabolomics–metallomics pipelines that respect spatial gradients. This is not merely descriptive microscopy; rather, it may be an important experimental requirement for testing the core hypothesis that B operates preferentially as an interface-level organizer, not as a uniformly distributed systemic factor.

### 7.3. Ecological Consequences Beyond Quorum Sensing Precedents

AI-2B remains a powerful mechanistic anchor because it shows, unambiguously, that B can be part of microbial signal identity. But the broader question is larger than AI-2: *what does B availability do to microbial ecology as a system?* The symbiotaxis framework suggests effects that may not be fully captured by taxonomy-first readouts: cooperation, niche stability, cross-feeding efficiency, spatial biogeography, and the resilience of community assembly after perturbation. Testing these predictions will likely require experimental designs that combine defined consortia or controlled complexity, carefully controlled B chemistry (form and accessibility), and spatially structured gut models that can quantify metabolite gradients and cooperation metrics, e.g., continuity of SCFA flux, stability of interaction networks, or recovery dynamics after stress. In other words, the field needs to move from “does B change who is there?” to “does B change how the community behaves and recovers?”.

### 7.4. Aging-Related Shifts in Boron Accessibility and Interface Fragility

The hypothesis becomes most consequential when placed in the context of aging, yet age-related changes in B biology remain underexamined. Across the lifespan, diet, digestion, MUC glycosylation patterns, epithelial permeability, and microbial functional capacity all shift, each of which could alter the balance between PAB and MAB. An important unresolved question is whether aging may be accompanied by a decline in interface-level B accessibility or a narrowing of the chemical contexts in which B can act, and whether such changes contribute to ecological volatility, barrier fragility, and inflammaging in susceptible individuals. Longitudinal cohorts and age-stratified studies, ideally coupled with measures of mucosal function and microbiome functional outputs, are needed to test whether B-related “architecture loss” is part of the aging trajectory [36,92].

### 7.5. Translational Design: Chemical Form, Matrix Effects, and Measurable Endpoints

Finally, translation is where many promising nutritional ideas become vague. If a substantial part of B’s biologically relevant effects is context-dependent and architecture-driven, then chemical form, colon delivery, and dietary matrix may be as important as total dose. Future nutritional and clinical studies may benefit from moving beyond intake-centric designs and incorporating measurements that directly speak to the model: B form and localization, microbiome functional outputs (SCFAs, bile acid profiles, redox-active metabolites), mucosal integrity and permeability markers, and integrative endpoints that reflect inflammatory–metabolic coherence rather than single biomarkers in isolation.

A practical way forward would be to prioritize (*i*) randomized interventions that compare PAB vs. MAB forms while monitoring mucosal integrity and inflammaging-relevant readouts, (*ii*) multi-omics integration (metagenomics, metabolomics, metallomics, transcriptomics) aimed at deriving quantitative indices of HM coherence, and (*iii*) precision-nutrition strategies that treat B accessibility as a contextual variable alongside fiber intake, polyphenol availability, and redox status. Together, these directions outline an agenda that connects chemical biology to microbial ecology and geroscience, while remaining grounded in what can actually be measured.

Ultimately, progress will likely require a conceptual shift: away from thinking of B primarily as a quantity to be ingested and toward thinking of B as a localized chemical capability whose biological significance emerges where interaction density, spatial structure, and signal persistence converge, exactly the levels at which HM architecture is built, maintained, and lost with age.

### 7.6. Closing Perspective

Collectively, these knowledge gaps highlight that the central challenge in B biology is not the absence of intriguing hypotheses, but the difficulty of resolving where, in what form, and under which spatial and ecological constraints B chemistry becomes biologically meaningful. Reframing B action in terms of interface-level architecture and communicative coherence shifts the focus from isolated molecular effects to emergent organizational properties of the HM system. Addressing these gaps will determine whether B-mediated symbiotaxis remains a compelling conceptual model or matures into a measurable, translationally relevant principle of aging biology.

## 8. Conclusions

This review presents a conceptual and hypothesis-generating framework rather than a definitive mechanistic model of B function in human physiology. Aging is increasingly understood as a systems-level process involving progressive loss of coordination across interacting biological networks. Within this view, the HM–mucosal interface may function as an important organizational hub, where disturbances in barrier integrity, microbial ecology, and local signaling environments can propagate into broader physiological dysregulation over time.

Against this background, we propose a reframing of B biology that moves beyond intake-centered and target-centered paradigms toward a spatially and chemically contextual interpretation of HM organization. By distinguishing PAB from MAB, we argue that the biological relevance of B cannot be reduced to systemic absorption or circulating levels alone. Rather, chemical form, spatial localization, and local accessibility may be critical determinants of function.

Within this framework, MAB is proposed as the B pool most likely to influence mucus-associated and microbiota-facing processes through reversible interactions with glycans, polyols, polyphenols, and selected signaling intermediates. However, the extent to which such effects operate in vivo in the human gut remains incompletely characterized. Accordingly, concepts such as B-mediated symbiotaxis should be understood as organizing and testable hypotheses, not as established physiological mechanisms.

The value of this framework lies in its capacity to generate experimentally tractable predictions. These include the expectation that B localization and speciation may influence mucus-associated organization, signal persistence, and microbial spatial patterning in ways that are not captured by total systemic B measurements alone. More broadly, the model suggests that relevant biological effects of B may be expressed less as large taxonomic shifts and more as changes in resilience-related properties, including reduced ecological volatility, improved barrier-associated order, and tighter coupling between microbial and host signaling processes.

Importantly, the relationships proposed here are likely to be bidirectional. Age-associated changes in mucus composition, glycosylation, microbial enzymatic activity, and dietary context may themselves reshape B speciation and accessibility, thereby modifying the chemical landscape of the HM interface. This reciprocal dynamic argues against a simple linear causal model and instead supports a context-dependent interpretation of B function in aging-associated DYS and inflammaging.

Overall, viewing B through the lens of molecular architecture and HM symbiosis offers a useful bridge between chemistry, microbial ecology, barrier biology, and resilience-oriented geroscience. The aim of the present review is not to position B as a singular determinant of healthspan, but to define a transparent and testable framework through which B-dependent chemistry may contribute to the maintenance of biological organization under conditions of age-associated fragility. Future work should therefore focus on resolving in vivo B speciation and localization, developing compartment-specific analytical approaches for MAB, and determining whether local B chemistry can measurably influence HM stability in aging-relevant settings.

## Figures and Tables

**Table 1:** Unique chemical properties of B as a driver of mucosal barrier integrity and inflammaging modulation.

B Chemical Property	Molecular Basis	Primary Effect on Mucosal Interface	Implication for Inflammaging and Aging Resilience
Lewis’ acidity (electron-deficient B)	trivalent B accepts electron pairs from hydroxyl donors	enables conditional, reversible interactions with glycan-rich matrices	stabilizes chemically fragile mucosal environments that progressively degrade with age
reversible covalent B–O bonding	dynamic borate ester formation sensitive to pH and ligand competition	supports adaptive molecular scaffolding rather than fixed complexes	buffers age-associated fluctuations in mucus chemistry linked to barrier breakdown and chronic inflammation
selective affinity for *cis*-diol motifs	cyclic ester formation with vicinal hydroxyls (MUCs, polyols, phenolics)	targets MUC glycans and dietary ligands at the gut interface	mechanistically links B to preservation of epithelial separation, a key determinant of inflammaging
ligand-dependent B speciation	interconversion between free BA and ligand-bound forms	concentrates B activity at interfaces rather than in plasma	explains disconnect between plasma B levels and barrier-associated aging outcomes
weak multivalency and network participation	numerous low-affinity interactions across glycan networks	modulates mesoscale mucus properties (viscosity, permeability, diffusion)	may limit microbial encroachment and immune activation associated with age-related dysbiosis
non-receptor, non- enzymatic mode of action	absence of classical signaling or cofactor roles	acts through structural modulation rather than pathway activation	aligns with the geroscience view that inflammaging arises from architectural failure, not single signaling defects
compatibility with polyphenol and polyol complexes	stabilization of dietary ligand assemblies	couples dietary phytochemicals to mucosal chemistry	supports dietary strategies that reinforce barrier function and reduce inflammatory tone with aging
plausible interaction with mucus glycan architecture	high density of *cis*-diols in O-glycosylated MUCs	enables microstructural tuning of mucus gel organization	direct relevance to age-associated mucus thinning, increased permeability, and immune–microbial contact

B: Boron; BA: Boric acid; MUC: Mucin; O: Oxygen.

**Table 2:** Operational distinctions between PAB and MAB.

Feature	PAB	MAB
primary location	blood and systemic tissues	intestinal lumen, mucus layer, host–microbe interface
dominant chemical forms	predominantly BA and small borates	ligand-bound, complexed, and/or matrix-associated B species
defining criterion	efficient epithelial uptake and systemic exposure	persistence and accessibility within luminal/mucosal microenvironments
typical measurement focus	plasma and urine (total B)	rarely measured directly; requires speciation-aware approaches (lumen/mucus-associated B, B–ligand complexes)
mode of action (dominant)	context-dependent systemic modulation (indirect)	interface-level structural and ecological effects (signal persistence, microstructure, niche organization)
relevance to aging biology	systemic markers and broad physiological endpoints	barrier integrity, inflammaging, DYS-related drift, community coherence
translational implication	dosing often optimized for systemic exposure	formulation/diet design optimized for interface localization and functional accessibility

B: Boron; BA: Boric acid; DYS: Dysbiosis; MAB: Microbiota-accessible boron; PAB: Plasma-accessible boron.

**Table 3:** Levels of organization potentially influenced by B-mediated molecular architecture.

Level of Organization	B-Linked Process	Functional Implication
molecular	reversible complexation with *cis*-diols	conditional stabilization of ligands and signaling intermediates
interface (mucus)	modulation of glycan-rich matrices and microdomains	barrier integrity, spatial separation, diffusion geometry
microbial community	changes in signal persistence and spatial distribution	coordination, cross-feeding efficiency, niche stability
ecological (network)	reduced volatility/improved recovery after perturbation	resistance to DYS and stress-induced collapse
system level	maintenance of host–microbiome coherence	lower propensity for inflammaging and functional decline

B: Boron; DYS: Dysbiosis.

**Table 4:** Conceptual links between B-mediated symbiotaxis and host resilience pathways (hypothesis-driven).

Interface-Level Feature (Symbiotaxis)	Primary Gut-Derived Mediators	Host Integration Nodes	Predicted Resilience Outcome
improved microbial coordination	SCFAs, polyamines, coordinated community metabolism	AMPK tone; mitochondrial adaptability	higher metabolic flexibility; reduced energetic “noise”
stabilized mucosal organization	lower permeability cues; reduced translocation; controlled proximity	NF-κB thresholding; immune calibration; inflammasome set-points	lower inflammaging probability; improved barrier-linked tolerance
conditioned redox microenvironment	redox-active metabolites; reduced oxidative bursts at the interface	Nrf2 responsiveness; redox buffering capacity	improved redox control; lower chronic stress amplification
more stable nutrient/signal gradients	bile acid profiles; indoles; consistent metabolite timing	SIRT1/NAD^+^ homeostasis; circadian–metabolic coupling	improved metabolic timing; stronger stress adaptation
reduced ecological volatility	stable niche structure; fewer abrupt community transitions	mTOR set-point regulation; growth–maintenance balance	lower maladaptive anabolism; improved maintenance programs

AMPK: Adenosine monophosphate-activated protein kinase; B: Boron; mTOR: Mechanistic target of rapamycin; NAD^+^: Nicotinamide adenine dinucleotide, oxidized form; NF-κB: Nuclear factor-kappa B; Nrf2: Nuclear factor erythroid 2-related factor 2; SCFAs: Short-chain fatty acids; SIRT1: Sirtuin 1.

## Data Availability

The original contributions presented in this study are included in the article. Further inquiries can be directed to the corresponding author.

## Abbreviations

The following abbreviations are used in this manuscript:

AI-2	Autoinducer-2
AI-2B	Autoinducer-2–borate
AMPK	Adenosine monophosphate-activated protein kinase
B	Boron
BA	Boric acid
B(OH)_3_	Boric acid
B(OH)_4_^–^	Tetrahedral borate species
dCACHE	Double calcium channels and chemotaxis
DCB	Diester chlorogenoborate
DNA	Deoxyribonucleic acid
DPD	4,5-Dihydroxypentane-2,3-dione
DYS	Dysbiosis
GI	Gastrointestinal
GM	Gut microbiome
HM	Host–microbiome
LC–MS	Liquid chromatography–mass spectrometry
MAB	Microbiota-accessible boron
MAFLD	Metabolic dysfunction-associated fatty liver disease
MAFs	Microbiota-accessible forms
MERA	Microbiome epigenetic rescue agents
mTOR	Mechanistic target of rapamycin
MUC	Mucin
MUC2	Mucin 2
NAD^+^	Nicotinamide adenine dinucleotide, oxidized form
NF-κB	Nuclear factor-kappa B
NMR	Nuclear magnetic resonance
Nrf2	Nuclear factor erythroid 2-related factor 2
O	Oxygen
PAB	Plasma-accessible boron
QS	Quorum sensing
RNA	Ribonucleic acid
SCFA	Short-chain fatty acid
SIRT1	Sirtuin 1
